# Dissociating Sensorimotor Recovery and Compensation During Exoskeleton Training Following Stroke

**DOI:** 10.3389/fnhum.2021.645021

**Published:** 2021-04-30

**Authors:** Nadir Nibras, Chang Liu, Denis Mottet, Chunji Wang, David Reinkensmeyer, Olivier Remy-Neris, Isabelle Laffont, Nicolas Schweighofer

**Affiliations:** ^1^Department of Biomedical Engineering, University of Southern California, Los Angeles, CA, United States; ^2^Euromov Digital Health in Motion, University of Montpellier, IMT Mines Alès, Montpellier, France; ^3^Neuroscience Graduate Program, University of Southern California, Los Angeles, CA, United States; ^4^Department of Mechanical and Aerospace Engineering, Anatomy and Neurobiology, University of California, Irvine, Irvine, CA, United States; ^5^Université de Brest, Centre Hospitalier Universitaire, LaTIM-INSERM UMR 1101, Brest, France; ^6^Montpellier University Hospital, Euromov Digital Health in Motion, Montpellier University, Montpellier, France; ^7^Division of Biokinesiology and Physical Therapy, University of Southern California, Los Angeles, CA, United States

**Keywords:** motor recovery, motor compensation, stroke, joint synergy, upper limb, neurorehabilitation, movement analysis

## Abstract

The quality of arm movements typically improves in the sub-acute phase of stroke affecting the upper extremity. Here, we used whole arm kinematic analysis during reaching movements to distinguish whether these improvements are due to true recovery or to compensation. Fifty-three participants with post-acute stroke performed ∼80 reaching movement tests during 4 weeks of training with the ArmeoSpring exoskeleton. All participants showed improvements in end-effector performance, as measured by movement smoothness. Four ArmeoSpring angles, shoulder horizontal (SH) rotation, shoulder elevation (SE), elbow rotation, and forearm rotation, were recorded and analyzed. We first characterized healthy joint coordination patterns by performing a sparse principal component analysis on these four joint velocities recorded during reaching tests performed by young control participants. We found that two dominant joint correlations [SH with elbow rotation and SE with forearm rotation] explained over 95% of variance of joint velocity data. We identified two clusters of stroke participants by comparing the evolution of these two correlations in all tests. In the “Recoverer” cluster (*N* = 19), both joint correlations converged toward the respective correlations for control participants. Thus, Recoverers relearned how to generate smooth end-effector movements while developing joint movement patterns similar to those of control participants. In the “Compensator” cluster (*N* = 34), at least one of the two joint correlations diverged from the corresponding correlation of control participants. Compensators relearned how to generate smooth end-effector movements by discovering various new compensatory movement patterns dissimilar to those of control participants. New compensatory patterns included atypical decoupling of the SE and forearm joints, and atypical coupling of the SH rotation and elbow joints. There was no difference in clinical impairment level between the two groups either at the onset or at the end of training as assessed with the Upper Extremity Fugl-Meyer scale. However, at the start of training, the Recoverers showed significantly faster improvements in end-effector movement smoothness than the Compensators. Our analysis can be used to inform neurorehabilitation clinicians on how to provide movement feedback during practice and suggest avenues for refining exoskeleton robot therapy to reduce compensatory patterns.

## Introduction

Individuals with stroke-induced loss of sensorimotor functionality in the upper extremity often experience some degree of improvements in the 6-month period after stroke. Such improvements have been observed in motor impairment (Broeks et al., 1999; Duncan et al., 2000), in motor function (Wolf et al., 2001; Yozbatiran et al., 2008; Kitago et al., 2013; Winstein et al., 2016), and in measures of end-point kinematics, such as movement smoothness, speed, or range (Rohrer et al., 2002; Van Dokkum et al., 2014; Leinenga et al., 2016; Schweighofer et al., 2018). Improvements in function and end-point kinematics can be due to true recovery, defined here as the ability to perform movements in the same manner as they were done before injury, or due to compensation, which occurs when the movements are performed in a new manner using alternate movement patterns, or both (Levin et al., 2009; Jones, 2017). Indeed, individuals post-stroke often learn to develop compensatory strategies during upper extremity movements, such as leaning forward (Roby-Brami et al., 2003; Bakhti et al., 2017) or elevating the shoulder or the elbow (Steenbergen et al., 2000).

In previous work with individuals in the sub-acute phase post-stroke (Schweighofer et al., 2018), we studied the changes in end-point smoothness of reaching movements in 3D space during 4 weeks of training with the redundant ArmeoSpring exoskeleton (Hocoma, Inc). We showed that changes in smoothness followed an initial fast phase, which we attributed to learning to control the device, and a slower phase that strongly correlated with reduction in overall upper extremity impairment, as measured by the Upper Extremity Fugl Meyer assessment (UEFM) (Fugl-Meyer et al., 1975). However, because the ArmeoSpring is a redundant system with more degrees of freedom (DOF) than those of the end-effector in external space, it was unclear to what extent these fast and slow improvements in end-point kinematics were due to true recovery or to compensation.

A possibility to study true recovery in arm movements is to limit reaching movements to the horizontal plane, while allowing only shoulder horizontal (SH) rotation and elbow rotation and constraining trunk movements. In this case, the arm is not redundant and no compensation is possible: only a single possible joint coordination pattern can be used to perform a desired movement in task space. Improvements in performance observed with this method can therefore be attributed to true recovery (Cortes et al., 2017). In particular, planar movements can be used to study the change in atypical joint couplings post-stroke, or “atypical synergies” (Fugl-Meyer et al., 1975; Reisman and Scholz, 2003; Ellis et al., 2005), between SH rotation and elbow rotation (Dipietro et al., 2007). During the initial recovery stage, patients typically lose independent joint control (Dewald et al., 1995). Later, they can move in any direction by regaining more independent control of joints (Brunnstrom, 1970). Using planar 2D movements, Dipietro et al. (2007) showed that an initial strong atypical coupling between horizontal shoulder rotation and elbow flexion/extension at inclusion decreased following robotic training.

To study compensation, we need to analyze the joint patterns made with a redundant arm. For instance, assuming that arm movements in 3D space made with the ArmeoSpring exoskeleton are restricted at the wrist, scapula, and trunk, there are four DOFs, and therefore one extra DOF: (i): arm flexion/extension; (ii) arm adduction/abduction; (iii) arm internal(medial)/external(lateral) rotation; and (iv) elbow flexion/extension (Maciejasz et al., 2014). Whereas the healthy motor system makes use of this redundancy to adjust joint coordination to optimize movements (Todorov and Jordan, 2002), the redundancy allows individual post-stroke to accomplish the reaching tasks despite the atypical synergies by recruiting other joints. Comparison of the changes in joint patterns during reaching between non-disabled controls and individuals post-stroke can thus shed light on compensation vs. true recovery.

Here, we therefore dissociated true recovery from compensations by analyzing both end-effector and joint kinematic data from a sub-group of participants who received 4 weeks of training with the ArmeoSpring, with two sessions per day. We examined data from ∼80 arm reaching tests, one before and one after each training session. In addition, we tested a group of 11 control participants who took part in 10 training sessions over 1 week, with 20 tests. We compared the evolution of joint coupling patterns over time for the stroke participants to the corresponding patterns for the control participants at the end of training. We hypothesized that we would identify two clusters of stroke participants: Recoverers, with joint coupling patterns that converge toward those of the control participants, and Compensators, with at least one joint coupling pattern that deviates from those of the controls.

## Materials and Methods

### Participants

We examined arm kinematic data obtained from a sub-cohort of participants from the experimental group of the REM-AVC clinical trial (NCT01383512), a multi-center RCT of mechanized arm therapy post-stroke (Rémy-Néris et al., 2021). The goal of this RCT was to evaluate the medico-economic benefits in post-acute stroke of 4 weeks of standard care and motor arm therapy with ArmeoSpring vs. standard care and self-rehabilitation. The inclusion criterion for the RCT were: age between 18 and 81 years, diagnosis of hemorrhagic or ischemic stroke 3 weeks to 3 months prior to inclusion, and a UEFM score between 10 and 40 points. Exclusion criteria included (1) pain in the affected shoulder >3/10 on a visual analog scale (VAS), (2) a Boston Diagnostic Aphasia Examination (BDAE) score ≤ 3 points, (3) fatigue or visual impairment that would prevent participation in an additional daily hour of therapy, and (4) incapability to sit independently. For the present study, we had access to ArmeoSpring kinematic data and to clinical data of 53 participants with a single stroke in the territory of the middle cerebral artery (MCA) (30 males, 19 females, 4 gender not available; 59.3 ± 13.9 years old; baseline UEFM 24.7 ± 9.1, final UEFM 37.2 ± 15.1, days since stroke 56 ± 21 days – all reported values are mean ± SD). UEFM scores, which were unavailable for three participants, were measured by trained physical or occupational therapists.

**FIGURE 1:**
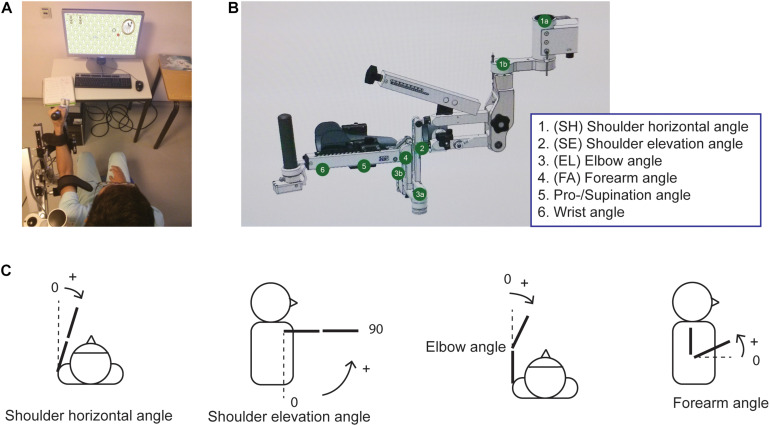
The ArmeoSpring device **(A)** Participant performing the Ladybug test. **(B)** The ArmeoSpring exoskeleton with the six possible angles. Note that in our study, movements in the wrist and pronation/supination joints were either immobilized or negligible and were therefore not analyzed. See text. **(C)** Illustration of angles demonstrating movements of the four different exoskeleton joints used in the analysis.

In REM-AVC, the participants were scheduled to receive training with the more affected arm on the ArmeoSpring, twice/day, 5 days/week, for a total of 40 sessions over a period of 4 weeks. Each training session lasted 30 min. A session consisted of several different video games (selected by the therapist and the patient in each session), and the ArmeoSpring vertical reaching tests “Ladybug” test (Figure 1A; see below for details) given at the beginning and at the end of each session, for a scheduled total of 80 tests (4 weeks × 5 days × 2 sessions × 2 tests). During these tests, joint and end-effector kinematics (see below) were recorded. We also analyzed the changes in UEFM from the week before training (initial UEFM) to the week following training (final UEFM).

In addition, to quantify normative reaching performance in both task and joint space, we recruited 11 young non-disabled participants (four females, 23.5 ± 2.0 years) as a control group for this study. These participants performed 10 video-game training sessions for 5 days. As with stroke participants, Ladybugs performance tests were also given before and after each training session (thus, for a total of 5 × 2 × 2 = 20 tests).

The part of the study including participants post-stroke was approved by the IRB of the University Hospital of Brest (CPP Ouest 6), Brest, France. The part of the study including non-disabled participants was approved by the IRB of the University of Montpellier, France. All participants read and signed an informed consent for participating in the study.

### The ArmeoSpring Device

The ArmeoSpring exoskeleton, based on the T-WREX device (Sanchez et al., 2006), has six DOF, summarized in Figure 1B. Two adjustable springs compensate for gravity at the upper arm and at the forearm, respectively (Cortés et al., 2016). Segment lengths can be adjusted to adapt to the user’s arm length. Users must move their arms to actively guide exoskeleton movement as none of the joints are assisted with motors. The user’s arm and forearm are attached to the exoskeleton with Velcro straps. The device records all joint angles and calculates the end-effector location in real-time through the exoskeleton’s forward kinematic model (developed by Hocoma, Inc.).

We note here that while there are similarities between ArmeoSpring DOFs and anatomical DOFs of the human arm, they are not identical. SH rotation in the device corresponds to anatomical shoulder adduction-abduction. Shoulder elevation (SE) on the device corresponds to anatomical shoulder flexion-extension. Elbow rotation on the device corresponds to anatomical elbow flexion-extension. However, forearm rotation on the ArmeoSpring is a combination of anatomical elbow flexion/extension, elbow pronation/supination and shoulder rotation. Finally, wrist rotation and wrist pronation/supination correspond to human movements. The ArmeoSpring joint rotations are demonstrated in Figure 1C.

### Exoskeleton Kinematic Testing

The Ladybug test (developed by Hocoma) is a two-dimensional pointing task in the frontal plane. Users were instructed to perform fast and accurate pointing movements to catch Ladybug targets that appeared sequentially on the screen by moving the cursor to target locations. The position of the cursor on the 2D screen depended on the position of the ArmeoSpring end-effector in the vertical plane. The sequence of target locations was fixed in each test. The user had to catch the Ladybug under a time-constraint (<10 s). Once a Ladybug was caught or the time limit was reached, the Ladybug disappeared, and the next Ladybug appeared at a new screen-location. The test had four possible difficulty levels, modulated both by the number of targets and by the workspace size. Each test session was separated into trials to parse out movement trajectories between consecutive targets. For participants in the stroke group, the therapist adjusted the test difficulty based on the participant’s performance and motivation. If a participant could catch more than 90% of the lady bugs in two consecutive sessions, then the therapist would increase the difficulty level. If the participant did not maintain this 90% success rate, the difficulty level was decreased. In the control group, difficulty was set to the highest level. Note that this study only analyzed end-effector trajectory and joint angle data from the Ladybug tests, but not from the training games in between the tests.

### Movement Data Processing

We used a second-order low-pass Butterworth filter (Butterworth, 1930) to filter the raw data (end-effector trajectory and joint angle trajectories) with a cutoff frequency of 5 Hz. We calculated end-effector velocities for each session by finding the derivatives of the end-effector displacement trajectories. We then calculated the number of peaks in the end-effector tangential velocity profiles for each trial. A peak was defined at any point where the velocity value was higher than the previous time-point and higher than the next time-point. End-effector performance in each test was assessed by the mean number of peaks using all trials in the test. We also measured smoothness in arm movements using a more robust metric, the spectral arc-length, which has been proposed as an alternative method to evaluate task space performance (Balasubramanian et al., 2012). We found that the mean number of peaks per trial and the mean spectral arc-length metric per trial were highly correlated in the stroke participant (*r* = −0.91). Thus, we reported the mean number of peaks to be consistent with our previous study (Schweighofer et al., 2018) (Note that task-space results slightly differ between this current study and this previous study because here we selected all trials, not only the successful trials).

We calculated the angular velocities across six joint angles by finding the derivatives of the respective joint angular displacements. We removed data from the first trial for every session because this trial often showed unrealistic high velocities caused by initial adjustment of cursor position at the beginning of sessions. We further removed data from 337 trials out of a total of 100,422 trials (0.3%) in which recording errors led to any of the following issues: the maximum angular velocities for any joint being higher than 10 rad/s at any point in a trial; the maximum angular velocities for any joint being less than 0.01 rad/s throughout a whole trial; or a trial lasting longer than 20 s. When more than half the data were removed for a session, we treated the session as a missing data-point for any further analysis. Nineteen out of a total of 3916 sessions (0.5%) were removed for this reason.

We analyzed angular velocity data instead of angular displacement data to remove any differences in starting positions across sessions from our analysis. Based on our visual observations of the participants performing the task, we suspected a negligible contribution of wrist movements to the overall movements. Indeed, the mean variance across the participants post-stroke for the angular velocities for the wrist and pronation/supination joints were much smaller than that of any of the other joints by a factor of at least 8. The mean session variance for angular velocities of the wrist and pronation/supination joints were 0.00163 and 0.00917 (rad/s)^2^, respectively, whereas the minimum mean session variances for any of the other joints were 0.0426 (rad/s)^2^. We therefore analyzed velocity data from four ArmeoSpring joints: SH rotation, SE rotation, Forearm rotation, and Elbow rotation.

### Sparse Principal Component Analysis of Joint Velocities of Control Participants

We then determined “healthy” normative joint coordination patterns by performing sparse principal component analysis (SPCA) on the four joint velocities concatenated from the last four test sessions performed by the young control participants (Zou et al., 2006). Previous studies have used Principal Component Analysis (PCA) for analysis of joint kinematics (Reisman and Scholz, 2003; Crocher et al., 2012; Van Kordelaar et al., 2012). SPCA is a variation of this method which allows for better interpretability for the principal components (PCs), as SPCA sets the weights of the joint velocities with small variance to be zero. Such a parsimonious model is obtained via a sparsity promoting regularizer.

Specifically, we pooled the velocity data from the last four sessions for each control participant (comprising all test data from their last day of training) and performed SPCA on the pooled joint angular velocities. Before performing SPCA, we scaled the joint velocities to unit variance to ensure joints with varying magnitudes of mean angular velocities were equally influential in determining PCs. We retained the number of PCs necessary to account for more than 90% of the variance in the pooled data. We then determined patterns of coordinated movements from the PC weights by observing which joint correlations explained the variance in the retained PCs. Joint velocities with weights of the same sign in a PC indicated that positively correlated velocities across these joints accounted for the variation explained by that PC. Joint velocities with weights of opposite signs in a PC indicated the same for negatively correlated velocities across those joints. For this analysis, we used the SPCA function in R (version 4.0.2) from the “*sparsepca*” package, which uses a combination of *lasso* (L1) and ridge (L2) regression (Erichson et al., 2020).

### Determining the Correlations of Interest Based on Sparse Principal Component Analysis

We examined the PC weights for all control participants and identified the SH-Elbow and SE-Forearm correlations as the two correlations of interest (see section “Results”). We therefore computed these two correlations of interest in the stroke group in each test using all the joint kinematic data. The Fisher z-transform was applied to distributions for both correlations to normalize the distributions. For the control group data, we then extracted 99% confidence intervals for each z-transformed distribution and defined these intervals as the respective control correlation ranges. Correlations within these ranges were defined as healthy joint coordination patterns. We defined any correlations outside these ranges as atypical joint coordination patterns.

### Nonlinear Mixed-Effects Models of Correlations of Interest and End-Effector Smoothness

We then modeled the changes in joint space performance (as measured by the z-transformed correlations of interest) and in end-point performance (as measured by movement smoothness) across test sessions for the participants in the stroke group via exponential mixed-effects models. We have previously shown that such mixed-effects models can account for the high variability across participants in performance, change in performance, and in responsiveness to therapy post-stroke (Park et al., 2016; Schweighofer et al., 2018).

Each correlations of interest was modeled with:

$$P{}_{\left(i;j\right)}=\left(A{}_{\text{i}}\times e^{-\frac{j}{\tau_{\text{i}}}}\right)+D{}_{\text{i}}+\varepsilon_{i,j},$$

where *P*_(*i*; *j*)_ is the estimated correlation for each participant (*i* = 1: 53) and each test session (*j* = 1: N, with N maximum = 86), A_*i*_ the approximate change in performance over the course of training for each participant, *D_i* the asymptotic value of performance reached at the end of training, τ_*i*_ the time constant for the participant’s rate of change, and ε_*i,j*_ is a normally distributed noise term. *P*_(*i*;*j*)_, *A*_*i*_, and *D*_*i*_ were expressed in units of z-transformed correlations, and τ_*i*_ was expressed in units of sessions and were all assumed to be normally distributed to reflect the inter-subject variability. The initial performance was estimated by *A*_*i*_ + *D*_*i*_.

We then modeled the change in the mean number of velocity peaks per trial for each session, using a double-exponential mixed effect model, as in our previous work (Schweighofer et al., 2018). In this model, the first exponential corresponds to a fast component, and second exponential to a slow component. Both joint and end-effector performance mixed models were fitted using the “*nlmefitsa”* function in MATLAB 2020a, which estimates the parameters using a stochastic Expectation-Maximization algorithm. We used Root Mean Square Error (RMSE) to measure the goodness of fit.

### Examining Change in Task Space Performance

Based on our previous study (Schweighofer et al., 2018), we expected that all stroke participants improved their task space performance over the 4 weeks of training. To confirm this, we performed two-sample unequal variances *t*-test between the initial and final values for the number of peaks per trial for all participants.

### Clustering Recoverers and Compensators

In contrast to task space performance, which was expected to improve for all participants, we hypothesized that changes in joint space performance would vary across participants. We clustered participants as “Recoverers” or “Compensators” based on the definition of true recovery: If over the 1 month of training both correlations of interests converged towards the mean correlations of the control subjects, we classified the participant as a “Recoverer.” In contrast, if at least one of the two correlations of interest deviated away from its respective control mean, we classified the participant as a “Compensator.” Specifically, for each participant, and both correlations of interest, we compared the difference between the fitted correlation and the control mean correlation at the end of training to the difference in the first trial. If there was a decrease in this difference, there was an improvement in the joint space during training. If a participant showed such improvements for both correlations of interest, we classified this participant as a Recoverer, with recovery in both task space and joint space. Alternatively, if at least one correlation of interest deviated away from its respective control correlation mean, we classified the participant as a Compensator, as the participant developed increasingly more atypical joint coupling(s) during training while still recovering in task space.

We then examined whether there were further differences between the two clusters (Recoverer and Compensator) by performing two-sample unequal variances *t*-tests for the following measures: initial UEFM score, final UEFM score, UEFM score change between pre- and immediately post-training, overall change in the mean number of peaks estimated from the double exponential fits, and time constants of the fast and slow components from the double exponential fits to the number of peaks. We checked normality using the Shapiro–Wilk normality test. If any of the measures was not normally distributed, we first log-transformed the distributions for both clusters. Normally distributed data were expressed as mean ± SD of the corresponding mean. Non-normally distributed data were expressed as median with interquartile range (IQR) (25% IQR, 75% IQR). Significance was set at the *p* < 0.05 level.

## Results

**FIGURE 2:**
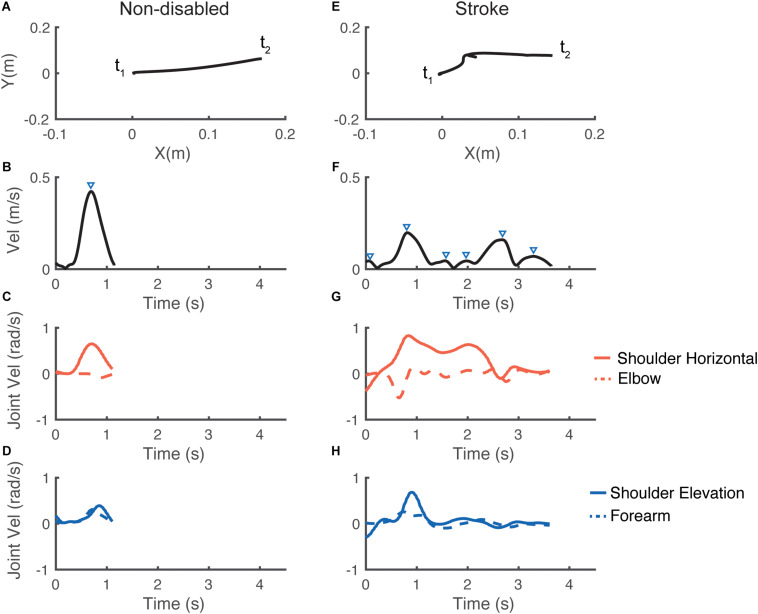
Representative performance for one individual in the control group (left panels) and for one individual in the stroke group (right panels) for the same trial. The first row **(A,E)** illustrates the trajectories of the cursor on the screen from *t*_1_: starting point to *t*_2_: end point. The second row **(B,F)** illustrates the corresponding end-effector velocity profiles; the triangles indicate the velocity peaks. The last two rows **(C,G)** and **(D,H)** show the corresponding joint velocity profiles. Note the longer movement duration and the less smooth performance for the participant with stroke, in both end-effector space and joint space.

Figure 2 shows examples of hand paths during similar trials, and corresponding velocity profiles in both task space (cursor velocity) and joint space (joint angular velocities) for a control group participant and for a participant with stroke (UEFM baseline = 34). Note that the control participant’s cursor trajectory was closer to being a straight line, and the velocity profiles showed fewer velocity peaks and were smoother than those for the stroke participant.

### Improvements in End-Effector Smoothness

**FIGURE 3:**
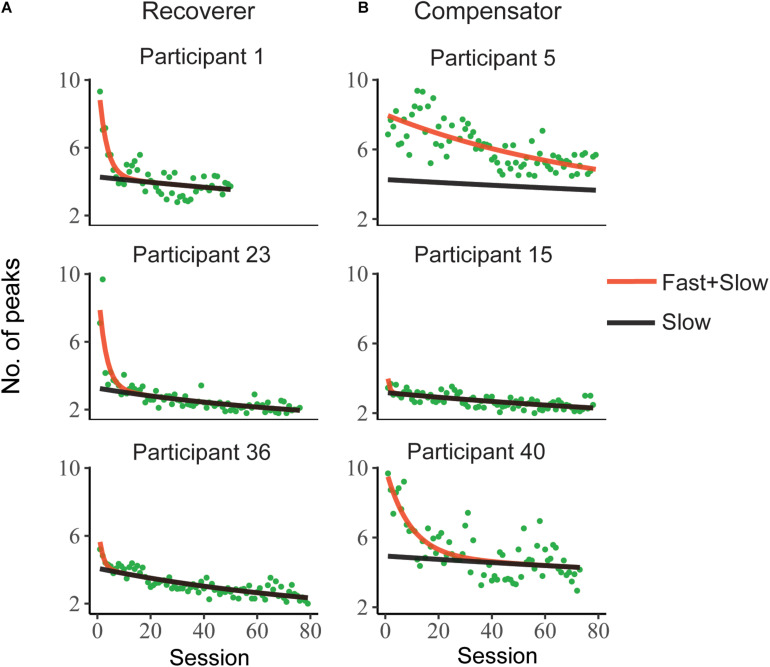
Representative evolution of the mean number of velocity peaks per trial during test sessions during training for six stroke participants. The red line represents the summed effects of fast and slow components in a double exponential mixed effect model. The black line represents the effect of the slow component for each individual. Note that the distinction between **(A)** Recoverers and **(B)** Compensators was made based on the analysis of changes in joint correlations (see text).

Stroke participants completed a mean of 74 ± 13 test sessions (range of 33–86) and 36% of them performed at least the scheduled 80 tests. All participants in the control group completed 20 test sessions. Figure 3 shows the mean number of velocity peaks per trial in each test and the double exponential model fits for six representative stroke participants (all participants’ data and model fits are in Supplementary Figure 1). The mean number of velocity peaks per trial improved for all participants post-stroke, with a decrease of 4.90 ± 2.41 peaks on average (as assessed by the model fit; two-sample unequal variances *t*-test [*t*(86) = 9.2, *p* < 0.0001]). Consistent with our previous study, the slow component was approximately linear, while the fast component decayed much faster (Schweighofer et al., 2018). The slow component had a median time constant of 222 tests (IQR = 153, 493) and the fast component a median time constant of 5.7 tests (IQR = 2.0, 11).

### Sparse Principal Component Analysis of the Control Participants

**FIGURE 4:**
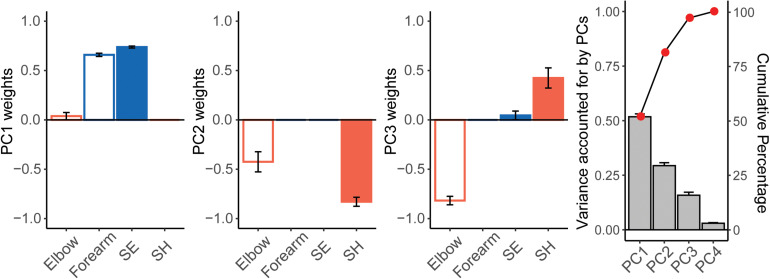
Results of the Sparse Principal Component Analysis (SPCA) for control participants. From left to right: weights from PCs 1, 2, 3, and variance accounted for by each PC (mean ± SE). We performed SPCA on each control participant, using the joint velocity data from the last four testing sessions (see text). Note how covariation of either the Forearm and SE joints (PC1) or of the Elbow and SH joints (PC2 and PC3) explain over 90% of the variance of the data.

Sparse principal component analysis was performed on pooled velocity data from the last four tests [last day of training from each control participant (see section “Materials and Methods” and Figure 4)]. PC1 explained 51.8 ± 4.3% of the variance, PC2 explained 29.4 ± 4.5% of the variance, and PC3 accounted for 15.9 ± 4.5% of the variance. PCs 1, 2, 3 explained more than 95% of the variance in joint velocities. The mean values of PC weights showed that PC1 was explained by positively correlated velocities in the Forearm and SE joints. PC2 was explained by positively correlated velocities of the SH and Elbow joints. In contrast, PC3 was explained by negatively correlated velocities of the SH and Elbow joints. In terms of anatomical movements, PC1 mostly corresponded to correlated velocities of shoulder flexion/extension with elbow flexion/extension for end-effector movement in the vertical plane. PCs 2 and 3 corresponded to correlated velocities of shoulder horizontal abduction/adduction and elbow flexion/extension for end-effector movement in the horizontal plane. The SE-Forearm correlation was therefore defined as the vertical synergy and the SH-Elbow correlation was defined as the horizontal synergy.

The z-transformed SE-Forearm and SH-Elbow correlation were 1.31 (99% CI = 1.18, 1.44) and 0.15 (99% CI = −0.06, 0.36), respectively. Z-transformed correlation values of 1.31 and 0.15 correspond to correlation values of 0.86 and 0.15, respectively. These values indicated that, when performing the Ladybug tests, the control participants showed strong coupling between SE and Forearm, i.e., the vertical synergy, while also showing decoupling between SH and Elbow, i.e., joints whose coupling would account for the horizontal synergy.

### Nonlinear Mixed-Effects Model for Joint Correlations

**FIGURE 5:**
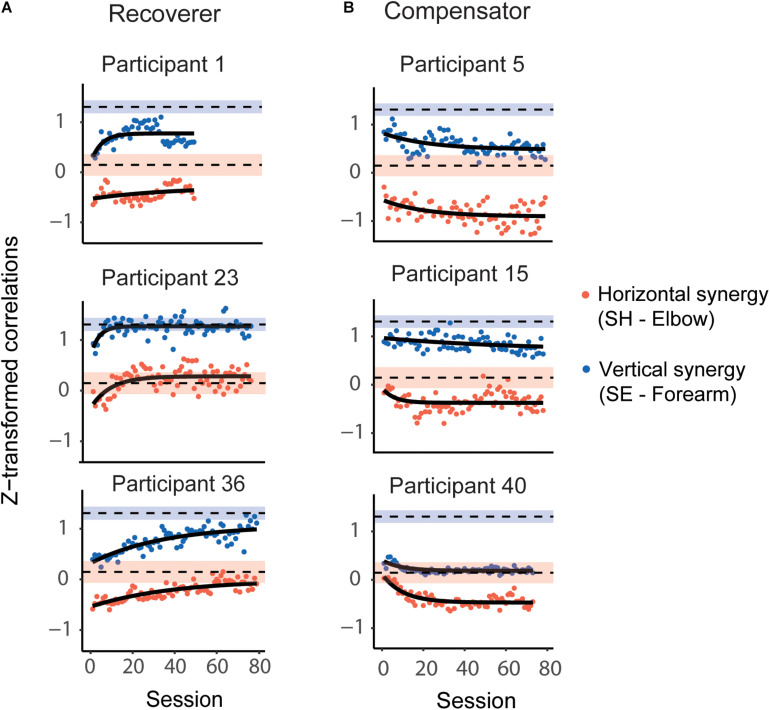
Representative evolution of the horizontal and vertical synergies, as defined by correlations in joint velocities, during Ladybug tests during 4 weeks of training; same post-stroke participants as in Figure 3. **(A)** Recoverers improved for both correlations, which were closer to the respective mean correlations in the control participants toward the end of training compared to the beginning of training. **(B)** Compensators deviated further from the respective correlation values for control participants for at least one of the correlations. The red dots represent the z-transformed SH-Elbow joint correlation; the blue dots represent the z-transformed SE-Forearm joint correlations. The black curves represent model fits. The blue shaded and red shaded areas represent the 99% CI range for the z-transformed SE-Forearm and SH-Elbow correlations, respectively, for control participants. The dashed black lines represent the mean values for the correlations for control participants.

Figure 5 shows the z-transformed SE-Forearm and SH-Elbow correlations for each session and the corresponding mixed-effect exponential model fits for six participants post-stroke (same participants as in Figure 3; all participants’ data and model fits are in Supplementary Figure 2). The models provided overall good fits to the participants joint (z-transformed) correlation data. RMSE was 0.13 (IQR = 0.12, 0.16) for the SE-Forearm correlation and 0.17 (IQR = 0.14, 0.19) for the SH-Elbow correlation.

Next, we compared the fitted values for SE-Forearm and SH-Elbow correlations for all stroke participants both at the beginning and the end of training to the healthy joint coordination patterns, i.e., the 99% CIs of the control participants’ correlations. At the beginning of the training, we found atypical SH-Elbow coupling, i.e., atypical horizontal synergy, in 38 participants, and atypical SE-Forearm coupling, i.e., atypical vertical synergy, in all participants except for one (52 participants). At the end of the training, we found atypical SH-Elbow coupling in 44 participants, and atypical SE-Forearm coupling in 47 participants. None of the participants were in the control correlation ranges for both synergies both at the beginning and end of training.

### Recoverers and Compensator Clustering

**FIGURE 6:**
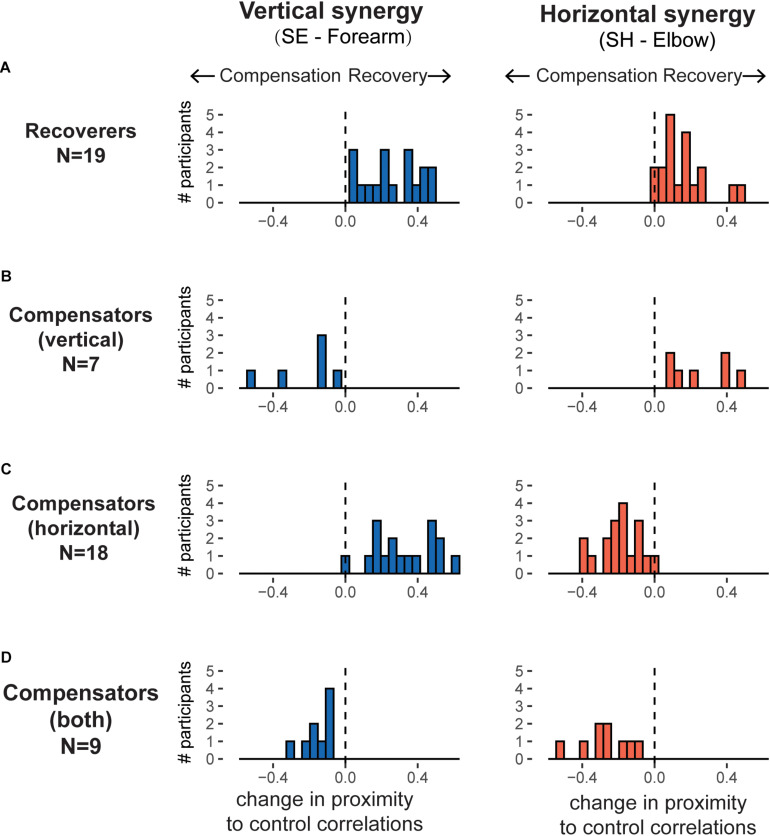
Histograms of evolution of the horizontal and vertical synergies, as defined by correlations in joint velocities, over training for the recovers and the three sub-clusters of compensators. For each panel, the horizontal axis represents the changes in correlation over training, with positive values indicating participants moving closer to control group synergies (recovery) and negative values indicating moving further away from control group synergies (compensation). The different patterns of change for the horizontal synergies (right panels) and for the vertical synergies (left panels) results in four possible clusters. **(A)** Recoverers (*N* = 19): improved in both synergies. **(B)** Vertical compensators (*N* = 7): improved in horizontal synergy but worsened in vertical synergy. **(C)** Horizontal compensators (*N* = 18): improved in vertical synergy but worsened in horizontal synergy. **(D)** Worsened in both synergies (*N* = 9).

We identified two clusters based on whether the participant’s final fitted correlation values were closer to the control mean at the end of training than at the beginning. Nineteen participants were in the Recoverer cluster as they showed improvements in both the vertical and horizontal synergies (Figure 6A). In contrast, 34 participants were in the Compensator cluster. Within the Compensator cluster, we identified three sub-clusters (Figure 6 and Table 1). The first sub-cluster comprised seven participants whose SH-Elbow velocity correlation moved closer to the control mean during training, but their SE-Forearm velocity correlation deviated further from the control mean, indicating that they improved in the horizontal synergy but worsened in the vertical synergy (Figure 6B). The second sub-cluster comprised 18 participants whose SE-Forearm velocity correlation moved closer to the control mean during training, but SH-Elbow velocity correlation deviated further from the control mean, indicating that they improved in the vertical synergy but worsened in the horizontal synergy (Figure 6C). The third sub-cluster comprised nine participants with both correlations deviated further from the healthy means, indicating that they worsened in both horizontal and vertical synergies (Figure 6D).

**TABLE 1:** Statistical evaluation of the Recoverer and Compensator clusters.

	Recoverer (*N* = 19)	Compensator (*N* = 34)	*t*	df	*p*-Value
Change in SE-Forearm correlations relative to control correlation mean^a^	0.27, IQR = (0.15, 0.4)	0.07, IQR = (−0.13, 0.34)			
Change in SH-Elbow correlations relative to control correlation mean^a^	0.15, IQR = (0.081, 0.19)	−0.18, IQR = (−0.26, −0.045)			
Initial UEFM	26.3 ± 10.3	22.8 ± 8.9	1.2	30	*p* = 0.240
Final UEFM	40.3 ± 16.4	35.6 ± 14.3	1.03	31	*p* = 0.310
Changes in UEFM^a^	14.1 ± 9.3	12.8 ± 9.5	0.47	36	*p* = 0.639
Change in number of peaks^a,b^	5.1, IQR = (4.2, 5.8)	4.7, IQR = (3.2, 6.2)	0.51	49	*p* = 0.617
Fast component^b^	2.8, IQR = (1.3, 5.9)	9.1, IQR = (3.2, 14.3)	−3.49	49	***p* = 0.00105**
Slow component^b^	200.2, IQR = (142.5, 291.0)	235.4, IQR = (152.8, 493.7)	−1.2	38	*p* = 0.240

*^*a*^Change refers to change between the beginning and the end of training. ^*b*^Indicate log transformed before performing unequal variance *t*-test. Bold text indicates that the p value was significant for inter-cluster differences.*

**FIGURE 7:**
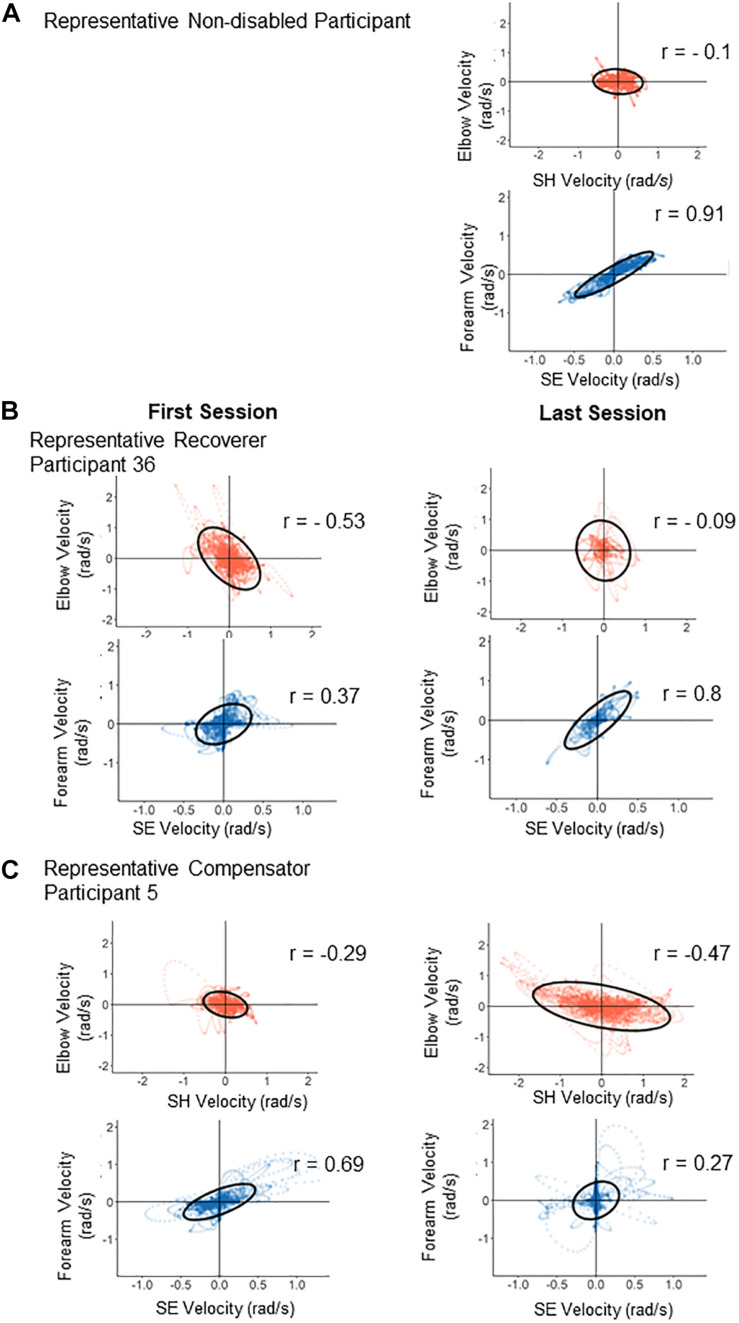
Illustration of changes in correlations from the first test (left panels) to the last test (right panels) for two representative participants. Each panel is a scatter plot of the velocities contributing to each synergy (red, horizontal synergy; blue, vertical synergy). **(A)** Typical control joint velocities from a control participant at the end of training: only the velocities contributing to the vertical synergy are strongly correlated. **(B)** The horizontal and vertical synergies of the Recoverer became close to those of the control participant pattern after training. **(C)** The horizontal and vertical synergies of the Compensator deviated further away from those of the control participant after training.

Figure 7 shows scatter plots of joint velocities for each synergy for a representative control participant at the last test session, and for representative Recoverer and Compensator participants in the first test and in the last test. For the control participant (Figure 7A), the SH-Elbow correlation was close to 0. The SE-Forearm correlation was close to 1, indicating that these two joints were strongly coupled. For the representative Recoverer (Figure 7B), the SE-Forearm correlation increased from *r* = 0.37 in the first test to *r* = 0.80 in the last test, indicating increased coupling. The SH-Elbow correlation decreased from *r* = −0.53 in the first test to *r* = −0.09 in the last test, indicating decoupling (Figure 7B). For the representative Compensator (Figure 7C), the SE-Forearm correlation decreased from *r* = 0.69 in the first test to *r* = 0.27 in the last test, indicating atypical decoupling. The SH-Elbow correlation decreased from *r* = −0.29 in the first test to *r* = −0.47 in the last test, indicating atypical coupling (Figure 7C).

Finally, we investigated whether the Recoverer and Compensator clusters showed differences in UEFM scores, in changes in the number of peaks, in motor learning as estimated via the fast component in the change number of peaks, or in reduction in impairment as estimated via the slow component in the change in the number of peaks (Schweighofer et al., 2018). There were no significant differences between the clusters in UEFM score changes [*t*(36) = 0.47, *p* = 0.639], initial UEFM scores [*t*(30) = 1.199, *p* = 0.240] and final UEFM scores [*t*(31) = 1.033, *p* = 0.310] (Table 1). However, we found significant differences in the log-transformed fast time constant [*t*(49) = −3.49, *p* = 0.00105]. The median time constant for the fast component was much lower for the Recoverers (2.8, IQR = 1.3, 5.9) than the Compensators (9.1, IQR = 3.2, 14.3), indicating that the Recoverers learned faster in the task space. In contrast, there were no significant differences between the log-transformed slow time constants [*t*(38) = −1.20, *p* = 0.240] or the change in mean number of peaks [*t*(49) = 0.50, *p* = 0.617].

## Discussion

We characterized changes in both end-effector performance and joint coordination patterns in individuals with post-acute stroke during 4 weeks of exoskeleton training. By performing a SPCA on joint velocities of control participants, we identified two main joint correlations, SH-Elbow and SE-Forearm. We defined the correlations as the horizontal synergy and vertical synergy, respectively. We then analyzed changes in these two correlations for all stroke participants via mixed-effect exponential models. By comparing the evolution of these two correlations to the corresponding correlations of control participants, we identified two clusters of individuals post-stroke. In the Recoverer cluster (*N* = 19), both correlations converged toward the respective mean correlations for control participants during training. In the Compensator cluster (*N* = 34), at least one correlation diverged from the respective means for control participants. Thus, Recoverers relearned how to generate smooth end-effector movements while developing synergies similar to those of control participants. In contrast, Compensators developed compensatory synergies dissimilar to those of control participants while still improving end-point performance. The compensatory patterns were varied, however, as they included atypical decoupling of the SE and Forearm joints, and atypical coupling of the SH and Elbow joints.

However, most stroke participants (51 out of 53), including most Recovers, did not reach the control correlation ranges at the end of training. This finding is consistent with previous studies that found stroke survivors exhibit atypical joint synergies compared to control participants (Cirstea and Levin, 2000). Although precise control in the gravity field remains difficult for people with stroke (Beer et al., 2007), the majority (37 out of 53) of the participants in our study developed more typical vertical synergies over the course of training. However, the horizontal synergy became more atypical for 27 post-stroke participants even with the counter gravity assistance of the ArmeoSpring. This atypical coupling of the SH and the elbow rotation joints is consistent with previous findings regarding synergy changes in chronic stroke patients (Dipietro et al., 2007).

There were no significant inter-cluster differences for initial and final UEFM scores, or UEFM score changes. This may suggest that while Compensators become as capable of independent joint control as Recoverers, the former learned to perform the task by learning new compensatory joint synergies. Thus, future studies should examine whether stroke participants who develop atypical compensatory joint synergies retain the capability of utilizing normal synergies. Additionally, since our study only included 4 weeks of training/test for early post-stroke, further extensive studies may be needed to track the long-term motor recovery for Recoverers and Compensators and determine if adopting compensatory strategies impair motor recovery assessed, for example, using UEFM.

As shown in our previous study, the change in the number of velocity peaks in task space can be modeled with a fast and slow component, with the fast component attributing to motor learning for using the exoskeleton (Schweighofer et al., 2018). Here, we showed that the time constant of the fast component was significantly smaller for the Recoverers than for Compensators (*p* = 0.00105). There were no significant inter-cluster differences for the slow component time constants (*p* = 0.240), which highly correlated with the reduction in impairment (Schweighofer et al., 2018). These results indicate that the compensators adapted more slowly to the dynamics of the device. A previous study has shown that learning a new muscle synergy pattern to perform a force-producing task is slower than learning muscle-force mapping similar to that of natural movement (Berger et al., 2013). Therefore, the slower learning rate in the task space that we observed for individuals in the Compensator cluster may be because compensators needed to form a new mapping between novel joint couplings and end-effector movement, which slowed down the motor learning rate in task space.

### Limitations

A first limitation of our study was that we did not use a motion capture system to collect the actual upper-limb kinematics during the clinical trial, but used a commercially available exoskeleton. An advantage to this set-up is the simplicity and reliability of data collection across the multiple sites who participated in the REM-AVC clinical trial. A disadvantage is that we recorded, and analyzed, exoskeleton angles, and there is no clear/direct mapping between these angles and anatomical angles for all joints. SH and SE were similar to anatomical shoulder abduction/adduction and shoulder flexion/extension, respectively, but the Elbow and Forearm angles were a combination of elbow flexion/extension and shoulder rotation. However, the exoskeleton joint angles allowed us to decompose the movements to joint synergies either aligned with or perpendicular to the gravitational field. Although the ArmeoSpring counteracted the forces of gravity, studies on how gravity affects movement synergy could benefit from our findings on how participants learned to perform different movements that are usually either affected by gravitational forces or independent of them (Beer et al., 2007; Ellis et al., 2009).

A second, and related, limitation is that we used a reaching test, the Ladybug test, for which the difficulty level was not constant, but was set for each session by the therapist based on the participant’s performance. Thus, the number and position of targets varied within and across the participants. To test whether difficulty level affects session joint correlations, we identified all participants (*N* = 6) who performed the Ladybug tests with the difficulty sequentially alternating between two difficulty levels for stretches of ten or more sessions, and performed linear mixed effect regression analysis between difficulty level and the two correlations of interest separately (in this analysis, we did not include participants for whom difficulty gradually changed since this would be confounded with time since stroke or motor recovery). We found no significant effects of difficulty level on the SH-Elbow (*p* = 0.6) or SE-Forearm (*p* = 0.7) correlations. Therefore, we did not adjust the correlation values by regressing out the effects of session difficulty. An additional reason for not adjusting correlation values was that it would remove the interpretability of correlation values, with corrected correlation values outside the −1 to 1 range lacking interpretability.

A third limitation is that our synergy and clustering analysis requires a motion capture system for the whole arm. However, while a gravity-compensating exoskeleton like the ArmeoSpring device may not be available to every rehabilitation center, a video-based motion capture system, including the inexpensive Kinect or equivalent systems, could be used to perform 3D joint level analysis in people post-stroke (Huber et al., 2015; Mobini et al., 2015; Bakhti et al., 2018).

A final limitation is that we compared the movement data of the stroke participants to those from a group of young non-disabled controls and not from a group of age-matched controls. We reasoned that the trajectories at the end of a week of training for young participants would be close to human “normative” trajectories. However, such movements may not be an accurate representation of what individuals post-stroke would have used prior to brain injury. Indeed, older non-disabled participants could use motor synergies different from young non-disabled adults during reaching tasks due to age-related changes in the neuromuscular system and the brain structure (Sleimen-Malkoun et al., 2013; Vernooij et al., 2016).

## Conclusion

To summarize, we found that while all participants with stroke recovered in the task space, only about a third relearned to move in joint space using patterns more similar to those of control participants during a month of kinematic training. These results are consistent with the proposal that post-stroke individuals become adept in new compensatory movements by performing repetitive movements through robotic training without receiving correction at the joint level (Crocher et al., 2012). The reason behind the emergence of different compensatory patterns could be subtle differences in residual force generation in individual muscles, and muscle weakness that are not detected by the UEFM (Jonkers et al., 2003; Mccrea et al., 2005). Another intriguing possibility is that individual differences in exploration in joint space could result in differences in learning compensatory movement patterns (Singh et al., 2016). Thus, additional studies are needed to understand how between-subject variability affects learning of different compensatory patterns. In any case, our analysis can be used to inform therapists on whether they should aim to reduce specific compensatory movements, and if so, what compensatory movements should be targeted. It can also be used to inform exoskeleton robots on where and how to add torques during therapy to affect compensatory patterns, if reduction of compensatory patterns are desired (Brokaw et al., 2011; Crocher et al., 2012).

## Data Availability Statement

The raw data supporting the conclusions of this article will be made available by the authors, without undue reservation upon request.

## Ethics Statement

The part of the study including participants post-stroke was approved by the IRB of the University Hospital of Brest (CPP Ouest 6), Brest, France. The part of the study including non-disabled participants was approved by the IRB of the University of Montpellier, France. All participants read and signed an informed consent for participating in the study.

## Author Contributions

NN and CL analyzed the data and wrote the manuscript. IL, DM, DR, and OR-N designed the experiment and edited the manuscript. CW collected and analyzed the data and edited the manuscript. NS designed the experiment, analyzed the data, and edited the manuscript. All authors contributed to the article and approved the submitted version.

## Conflict of Interest

DR receives royalties from UC Irvine for a patent licensed to Hocoma related to ArmeoSpring and has an equity interest in Hocoma. The terms of this arrangement have been reviewed and approved by the University of California, Irvine in accordance with its conflict of interest policies. The remaining authors declare that the research was conducted in the absence of any commercial or financial relationships that could be construed as a potential conflict of interest.
