# Supplementary material for: Dissociating Sensorimotor Recovery and Compensation During Exoskeleton Training Following Stroke
Source: Front Hum Neurosci. 2021 Apr 30;15:645021. doi: 10.3389/fnhum.2021.645021 (PMC8120113; doi:10.3389/fnhum.2021.645021)

**Supplementary Figure 1- Double exponential NLME fits for mean number of velocity peaks for all participants with stroke**

**
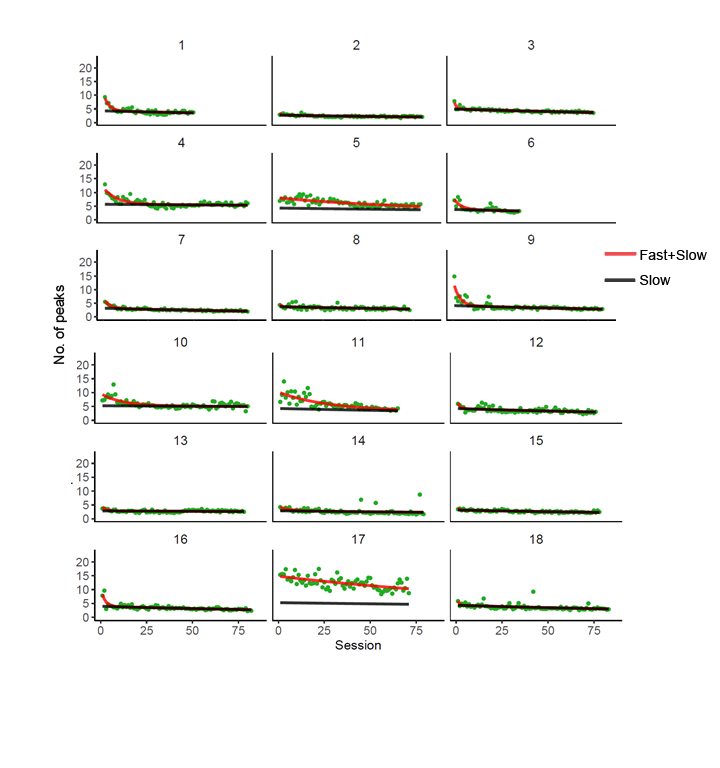
**

**
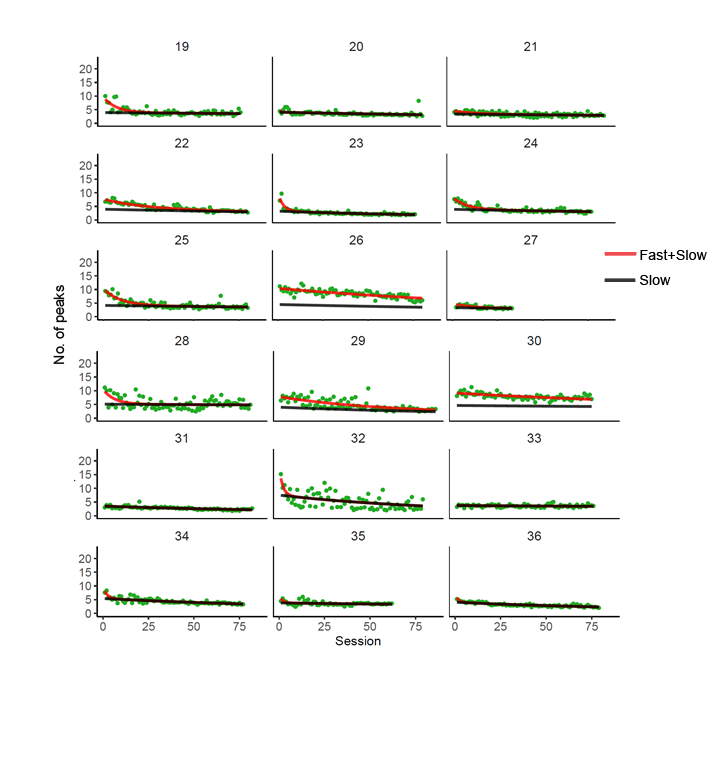
**


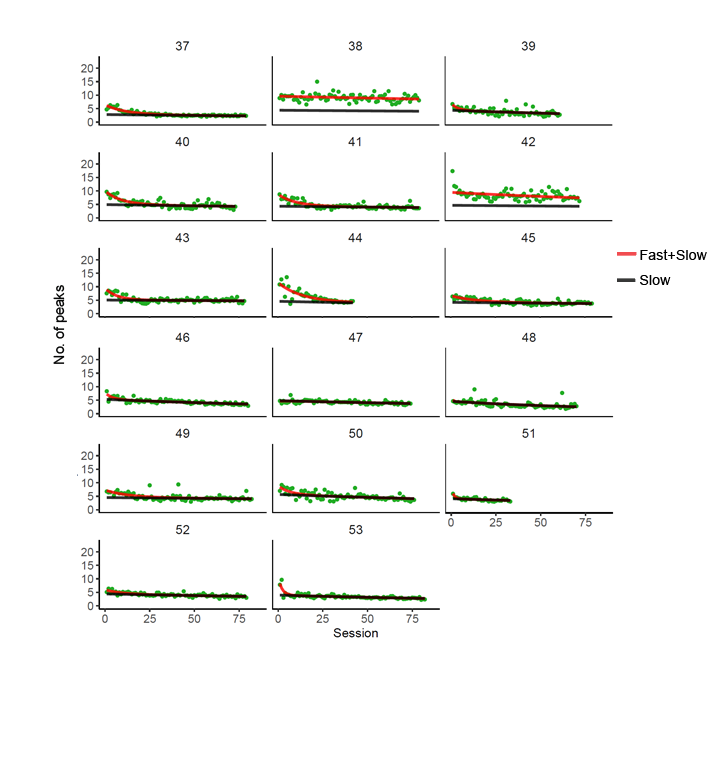


**Supplementary Figure 2**

**NLME exponential fits for SH-Elbow and SE-Forearm correlations over training for Recoverers**

**
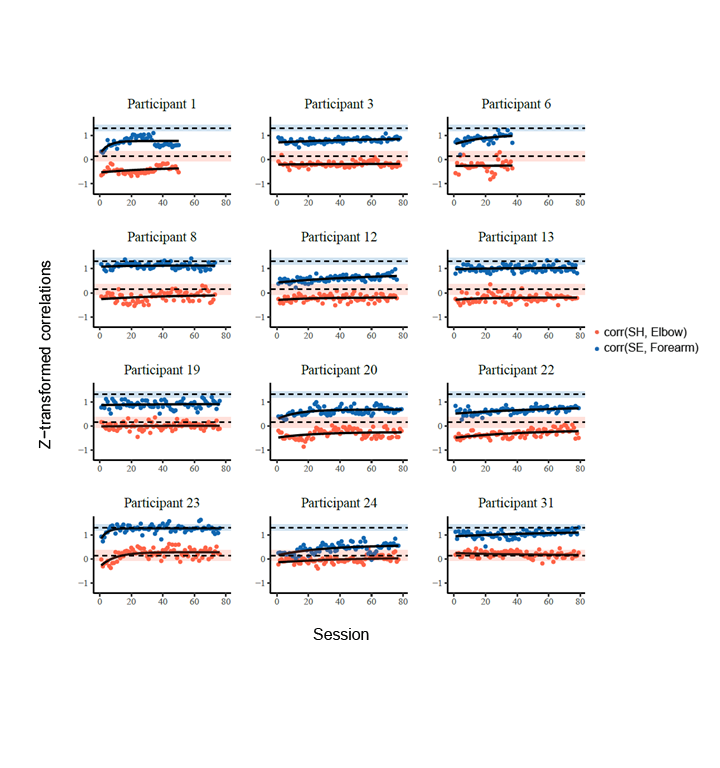
**


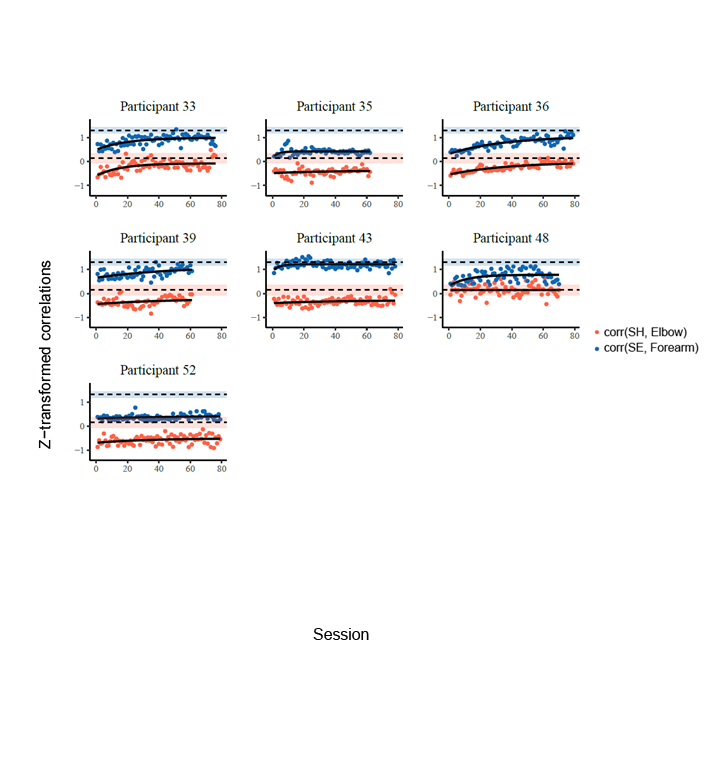


**NLME exponential fits for SH-Elbow and SE-Forearm correlations over training for Compensators**

**
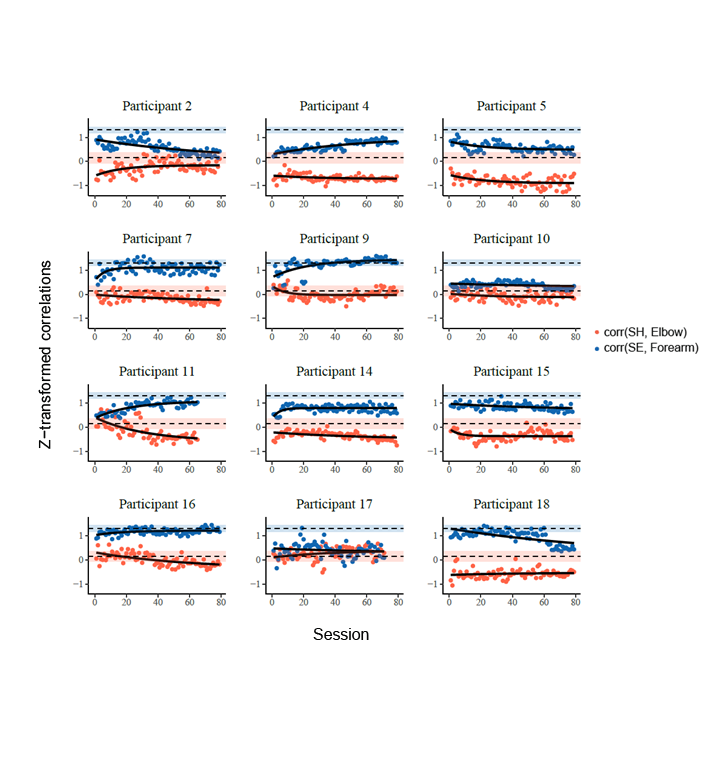
**


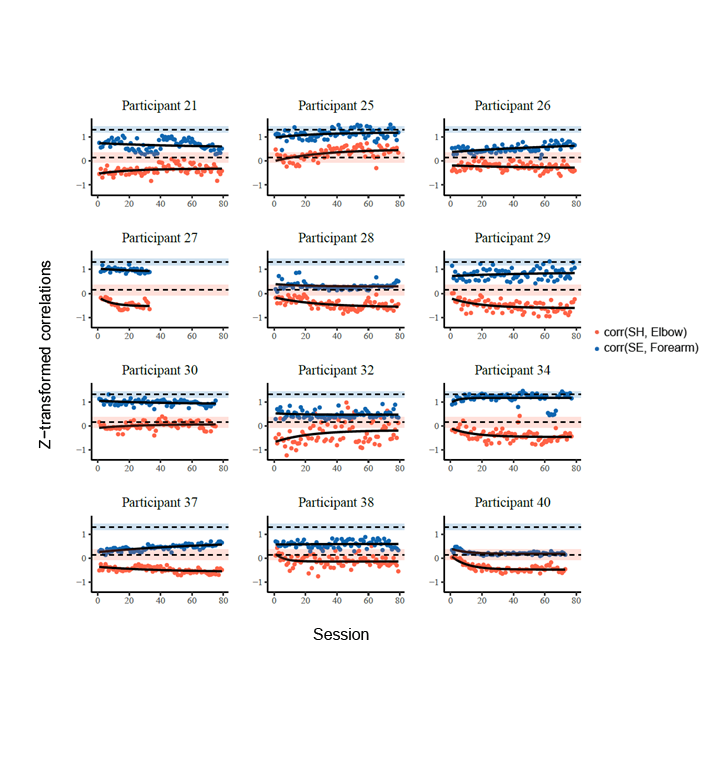

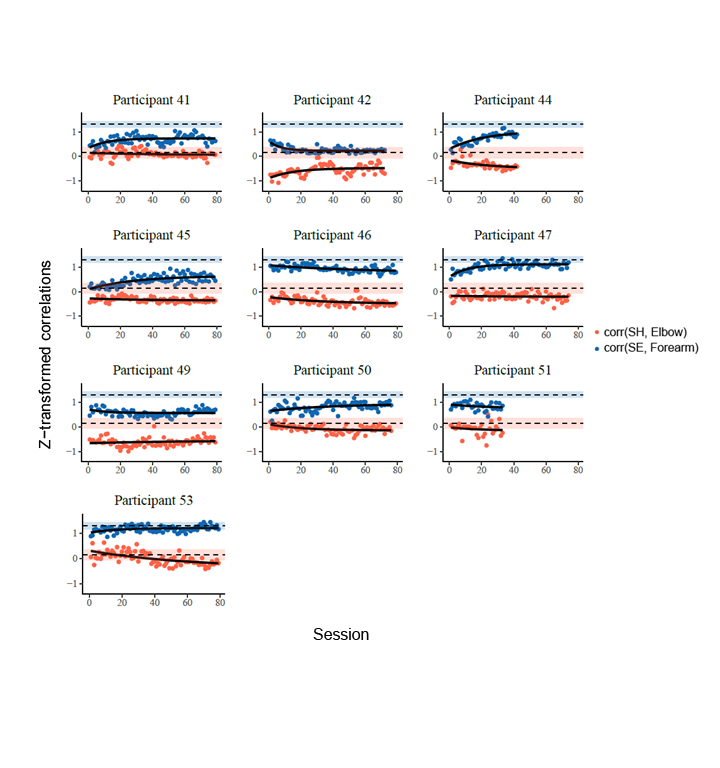

Supplement: Supplementary file 1 [file Table_1.DOCX]
